# Clinician and patient perspectives on meaningful Parkinson's disease impacts for digital assessment

**DOI:** 10.3389/fneur.2026.1677680

**Published:** 2026-03-20

**Authors:** Andrew Pearlmutter, Robert Ellis, M. Judith Peterschmitt

**Affiliations:** 1 Research and Development, Sanofi, Cambridge, MA, United States; 2 Koneksa Health, New York, NY, United States

**Keywords:** clinician and patient perspective, digital endpoint instrument, Parkinson's disease, Parkinson's disease functional impacts digital instrument (PD-FIDI), smartphone app, wearable

## Abstract

**Background:**

Novel clinical outcome measures that accurately track Parkinson's disease (PD) progression are required to enhance patient care by improving physician decision-making and optimizing clinical trial design. The Parkinson's Disease Functional Impacts Digital Instrument (PD-FIDI) is a smartphone-app and wrist-worn wearable-based digital endpoint instrument designed to remotely measure PD functional impacts, including severity, progression, and functional changes.

**Objectives:**

Evaluate clinician- and patient-perspectives on the most relevant and meaningful aspects of PD to incorporate into the PD-FIDI.

**Methods:**

An advisory panel of eight clinical experts guided a literature review of qualitative studies reporting patient perspectives on the everyday impacts of PD. Clinician perspectives were acquired from the same expert panel. The perspectives of people with PD were gathered through an online survey (*n* = 189 people with idiopathic PD; *n* = 13 people with PD and *GBA1* mutation). Clinician- and patient-perspectives were summarized through a meaningfulness classification scheme which scaled (low, medium, or high) the reported PD functional impacts.

**Results:**

Clinicians and patients considered a subset of motor (tremor, rigidity/stiffness, bradykinesia, global motor function, and hand and mobility difficulties) and non-motor (depression and fatigue) PD aspects to be most bothersome and meaningful if improved.

**Conclusions:**

PD aspects identified as highly meaningful were incorporated into the PD-FIDI's design where technically viable, operationally feasible, and safe. Results support the evaluation of the PD-FIDI in an ongoing clinical study which will determine the instruments' analytical and clinical validity and usability.

## 1 Introduction

Parkinson's disease (PD) is a progressive neurodegenerative condition characterized by motor and non-motor symptoms (1). While advances have been made in managing PD motor symptoms, little progress has been made in identifying therapies that can slow the rate of disease progression (2). Assessment of disease-modifying therapies may be hindered by limitations in clinical outcome measures that are subjective, time-consuming, and burdensome to complete, and do not sufficiently assess the patient in their daily living setting where symptoms may present differently than in the clinic (3–8).

Several PD symptoms and severity measures exist. Notwithstanding a recently proposed biologic staging system that integrates biomarkers, (9) these measures are solely based on clinical assessments and include the Hoehn and Yahr (H&Y) Scale (10), the Unified PD Rating Scale (UPDRS), the Movement Disorder Society-UPDRS (MDS-UPDRS) (11), and the 39-item PD questionnaire (PDQ-39) (12). However, each scale has documented limitations. The H&Y Scale typically requires a long period of time to observe a change in a patient's score (13), and may not adequately capture non-motor disease impacts and more subtle variations in disease severity (10). Although the UPDRS has been the most widely used PD clinical outcome measure in clinical trials for decades, it was developed when PD was thought to be a mainly motor disease, and incapacitating non-motor symptoms such as constipation, fatigue, and impaired sleep were inadequately captured (11). The updated MDS-UPDRS addresses some of these limitations but requires patients to visit clinics (11). Furthermore, clinician-raters are required for the MDS-UPDRS and other clinical scales, which is a source of measurement error in clinical trials because it limits the frequency of repeated measurements and is subject to inter- and intra-rater variability (3, 14, 15). The PDQ-39 is the most widely used patient-reported rating scale, though may not be suitable to detect small but clinically relevant changes in people with early PD (16, 17).

The availability of more accurate, reliable, and objective measures of PD functional impact could improve clinical trial conduct and clinical care while reducing patient and clinician measurement challenges. In the future, this information is expected to be captured partly by digital devices, including wearables and smartphones, which permit continuous monitoring in contrast to the snapshot provided by conventional clinical assessment (4, 5). While initial digital measures have been developed and tested (18, 19), the US Food and Drug Administration (FDA) has not yet, to our knowledge, approved a digital biomarker as a PD digital endpoint except for Neuron23 (NCT06680830). FDA guidance on patient-reported outcomes (20) and digital health technologies (21) indicates that the content of novel digital endpoint outcome measures must be validated such that the assessed disease characteristics and impacts are relevant and meaningful to patients' everyday lives.

**Figure 1:**
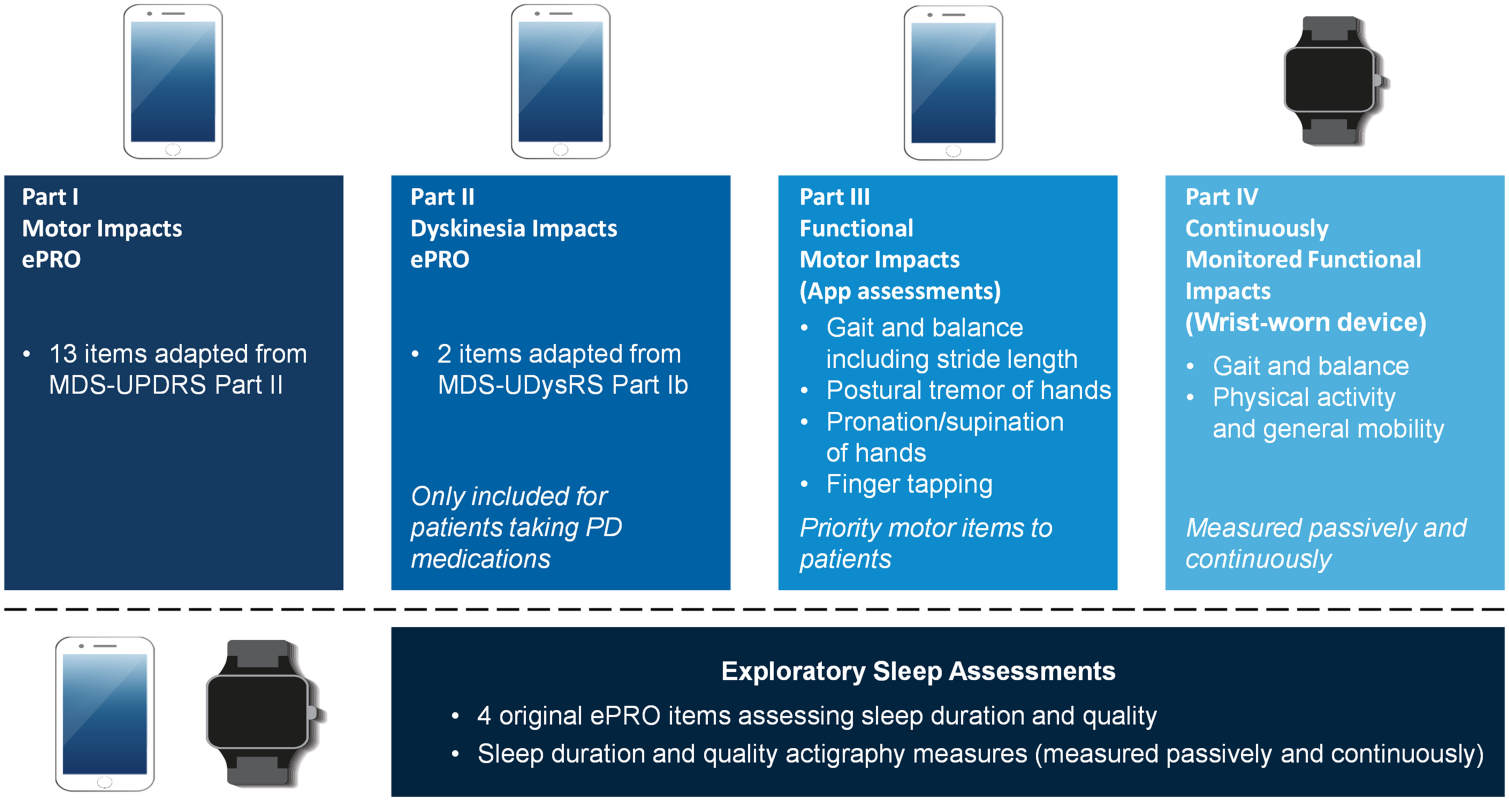
Overview of the PD-FIDI. The PD-FIDI includes an iPhone application with electronic patient-reported outcomes (ePROs) and functional assessments, plus a wrist-worn actigraphy device (ActiGraph GT9X). The two items adapted from MDS-UDysRS Part Ib were Question 4 (Eating Tasks) and Question 9 (Walking and Balance), and were selected based on review of dyskinesia-related endpoints for ongoing PD clinical trials. MDS-UDysRS, Movement Disorders Society-Unified Dyskinesia Rating Scale; MDS-UPDRS, Movement Disorders Society-Unified PD Rating Scale; PD, Parkinson's disease.

In parallel to Roche and others, we developed a fully digital PD measurement tool focused on everyday PD functional impacts and experiences: the Parkinson's Disease Functional Impacts Digital Instrument (PD-FIDI). The PD-FIDI uses an iPhone application and a wrist-worn device and includes four parts (Figure 1). Part I is an app-based electronic patient-reported outcome (ePRO) assessment of motor impacts that includes 13 items adapted from Part II of the MDS-UPDRS. Part II is an app-based ePRO assessment of dyskinesia impacts that includes two items adapted from Part Ib of the Movement Disorders Society-Unified Dyskinesia Rating Scale (MDS-UDysRS). Part III contains four app-based functional motor impacts assessments derived from mPower, a PD application built using Apple's open-source ResearchKit™ (22–24). Part IV includes continuous monitoring of gait and balance as well as physical activity and mobility using a wrist-worn activity tracker (ActiGraph GT9X). Additional sleep assessments being measured by the PD-FIDI device include four ePRO assessments and ActiGraph-based measures.

Our target population was mild to moderate stage PD (H&Y stages 1–3), with either idiopathic or *GBA1* variant PD. As part of our comprehensive approach to develop the PD-FIDI, we sought to identify the most relevant and meaningful aspects of PD to incorporate into this novel digital endpoint instrument with clinician- and patient-focused concept development research. This research was conducted in 2018–2019 and was guided by FDA feedback on the PD-FIDI obtained through a Type C meeting. The research methods are in line with the FDA's 2022 guidance on methods to identify what is important to patients (25) and included background research in the form of a review of relevant qualitative literature, clinical expert meetings, and an online patient survey.

## 2 Methods

### 2.1 Literature review

To inform the patient-focused concept development research, a literature review was performed in consultation with an advisory panel of PD clinical experts (see Acknowledgments). Of the articles reviewed, two publications directly evaluated which PD aspects matter most in patients' daily lives and functionality (26, 27).

Politis et al. (26) asked 265 people with early-stage (< 6 years since diagnosis) and advanced-stage (≥6 years) PD to identify the three most troublesome PD symptoms they had experienced in the last 6 months. Troublesome symptoms were identified by content analysis and weighted according to their ranking (i.e., 3 points were applied to the most troublesome symptom, 2 points to the second most troublesome symptom, and 1 point to the third most troublesome symptom). Weightings for each symptom were summed across patients, stratified by disease stage (26).

Deane et al. (27) surveyed 1,000 people with experience of PD, including 600 people with the condition, to determine shared views on the key future research priorities for PD management. Their study focused on identifying the top 10 uncertainties that should be prioritized in future PD research (27).

The articles by Politis et al. and Deane et al. were reviewed alongside a third article of relevance by Bonner et al., which describes research conducted by an author of the present article (MJP) in parallel with the qualitative research described in this report (26–28). Bonner et al. performed concept elicitation interviews to qualitatively understand the perspectives of people with PD regarding which symptoms and related aspects everyday of living were most important and meaningful to improve (28). They interviewed 20 people with PD (*n* = 15 people with PD and *GBA1* gene variant [*GBA1*–PD] and *n* = 5 people with idiopathic PD [iPD]) in a 2-h, semi-structured format. Participants were asked scripted questions about the three PD aspects they considered most bothersome and, separately, most important to treat. Conditional “concept probing questions” were asked if clarification was required.

### 2.2 Clinical advisory board meetings

An advisory panel of eight PD clinical experts (see Acknowledgments) was convened to solicit qualitative input on which functional impacts clinicians believe to be most clinically relevant and meaningful to PD patients. The initial panel meeting took place in January 2019 and used a hybrid in-person/virtual format. A follow-up meeting took place approximately 1 month later to share and validate key concept development research findings with the experts.

Both discussions were moderated to encourage the full participation of all panel members but were otherwise open-ended in format. Meeting minutes were recorded, and experts had the opportunity to review and provide feedback to ensure transparency.

### 2.3 PD patient online survey

To further understand which PD symptoms, matter most to patients we conducted an online survey of 202 people with PD in 2019. The survey contained both qualitative free-response and quantitative rating and ranking questions (Table 1) to identify which everyday functional PD aspects (Supplementary Table 1) most negatively impact patients, and which would be most meaningful to improve. Free responses were content-analyzed to detect systemic and category-based patterns in the PD functional aspects mentioned. The survey was deployed as online survey link through survey provider distribution channels. The survey was conducted by a third-party vendor, was not sample sized for a specific hypothesis, and was based on convenience sampling reflecting the maximum number of recruitable respondents within the predefined study timelines. The survey was targeted at people with documented diagnosis of Parkinson's disease, either idiopathic or carrying *GBA1* mutations. This population matches and continues prior work (28). However, in addition to mild and moderate stages of disease (H&Y 1–3), we expanded the inclusion criteria to patients with more advanced stages (H&Y 4–5).

**Table 1:** Questions included in the PD patient online survey.

**Question**	**Response type**
Please tell us how Parkinson's Disease has most negatively changed your everyday life. Which day-to-day experiences and activities have been most negatively affected, and how?	Open-ended, free text
If a new clinical drug could meaningfully improve one day-to-day experience or activity that Parkinson's Disease has most negatively affected in your everyday life, what would it be and why?	Open-ended, free text
Over the last week, did you experience any Parkinson's-related difficulties with the following experiences or activities? If so, how much did those difficulties negatively affect you? Select the best-fitting option.^a^	Rating^b^
If a new clinical drug could meaningfully improve one experience or activity that Parkinson's Disease has negatively affected in your everyday life which would it be? Please select one item from the list below.^c^	Ranking

^a^Question was shown five times, with five randomly generated lists of eight everyday functional difficulties shown on each page.

^b^Participant selected one of the following options: I did not have difficulties with this; I had difficulties with this but they DID NOT affect me; I have difficulties with this that SOMEWHAT affected me; I had difficulties with this that SIGNIFICANTLY affected me.

^c^Question was shown after each rating question along with the same randomly generated list of eight PD functional difficulties.

### 2.4 Comparison of clinician- and patient-perspectives

To determine which functional aspects of PD are viewed as most meaningful by both clinicians and patients, qualitative data from clinical advisory board meetings and the online survey were compared using a meaningfulness classification scale with three levels (low, medium or high). For the clinical advisory board meeting, a PD functional aspect was classified as low if it was not mentioned, medium if it was mentioned but never explicitly discussed as highly face valid or important, or high if it was explicitly discussed as highly face valid and important. For the online survey, a PD functional aspect was classified as low if it was consistently one of the least impactful and meaningful to improve across all relevant survey questions, medium if it was of average impact or meaningfulness to improve in all or some of the relevant survey questions, or high if it was consistently one of the most impactful and meaningful to improve across all relevant survey questions.

Sanofi led and funded the development of the PD-FIDI tool, and was involved in study design, tool development, patient surveys, and in the preparation of this manuscript. Sanofi commissioned Koneksa Health as the technology vendor, and convened an advisory panel of clinical experts.

## 3 Results

### 3.1 Literature review

Politis et al. found that in early-stage PD, motor symptoms such as slowness, tremor, and stiffness tended to be the most bothersome, scoring 112, 101 and 76 respectively. Slowness and tremor were identified as the primary bothersome symptoms for 32.6% and 29.3% of patients respectively. Stiffness, ranked third and reported by 26.1% of patients, was the leading secondary bothersome symptom. Pain was ranked fourth and was the leading bothersome non-motor symptom in early-stage PD. Additional significant bothersome symptoms included loss of smell/taste, issues with mood, handwriting difficulties, bowel dysfunction, sleep disturbances and loss of appetite, ranked fifth to tenth respectively (26).

In advanced stage PD, ineffective medication response, mood issues, and drooling were the most bothersome, scoring 115, 96, 85 respectively. Wearing-off phenomenon and increased jerking were the most prevalent issues for 63% and 22% of patients respectively, under medication category. Tremor and frequent falls were the only classical motor symptoms to appear in the the top 10 list for advanced stage PD, ranking fifth and ninth respectively. Additional significant bothersome symptoms included issues with bowel and urinary function and changes in weight/appetite and were ranked seventh, eighth, and tenth respectively (26).

Deane et al. used their survey data to produce a list of the top 10 priority areas for PD research (Supplementary Table 2) (27). An evaluation of these priority areas implies that people with PD prioritize improving difficulties relating to gait and balance, mental health, dyskinesias, tremor, cognition, sleep, dexterity, and urinary problems (27).

In the study by Bonner et al. (28), all 20 participants had mild to moderate PD (i.e., Hoehn and Yahr stages 1–3). The mean age of participants was 66 years (range 46–80 years) and there was an equal gender distribution (28). Fatigue, tremor, and memory loss were most frequently (≥90% of patients) selected as among the three most bothersome symptoms or most important symptoms to treat (Supplementary Table 3).

### 3.2 Clinical advisory board meetings

Clinical expert panel members highlighted the importance of assessing face valid PD aspects (i.e., those which have a clear and direct impact on patients' lives, such as physical activity level and gait quality/speed impacts). In addition, the expert panel emphasized the importance of measuring stride length and variability based on previous research relating these parameters to disease severity and patient perception of functional disability (29).

Panel members also recommended the inclusion of everyday functional aspects (e.g., hand tremor and global motor function) in the digital endpoint instrument because amelioration of these functional aspects would clearly demonstrate that a therapy is positively impacting people with PD.

Additionally, panel members suggested assessing cognitive impairment (memory and executive decision) and psychiatric impacts (delusions, hallucinations, anxiety, and depression) if feasible. OFF-state dystonias and difficulties falling and staying asleep were also noted as relevant.

### 3.3 PD patient online survey

This online survey was conducted across 39 states in the US. A total of 202 patients with documented PD were consented and enrolled, including 189 with iPD and 13 with *GBA1*-PD. Of note, 61% of participants reported having received genetic screening testing and/or genetic counseling prior to their participation in the survey. Survey participants had a mean age of 49 years, 59% were male, 23% had partial or full Ashkenazi Jewish ancestry (a population in which *GBA1* variants are particularly prevalent) (30), 27% were non-white; 84% were H&Y stages 1–3 (early- to moderate-stage; Supplementary Table 4), and 16% were H&Y stages 4 – 5 (late-stage). Patients receiving symptomatic therapy for PD were asked if they experienced ON & OFF states, and if they experienced worsening of ON-state dyskinesia, or worsening of tremor symptoms the week prior to their participation in the study.

**Figure 2:**
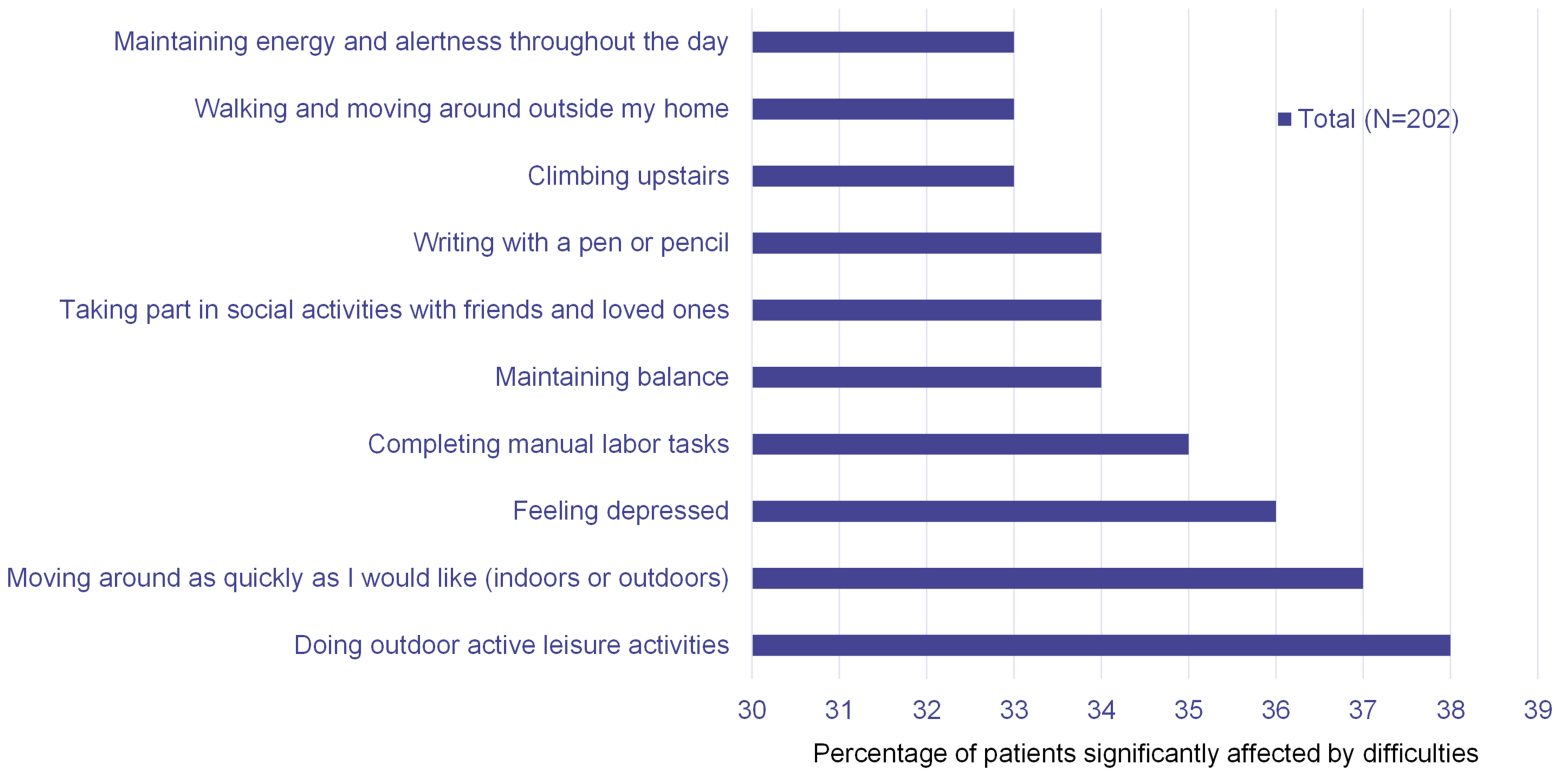
Patient online survey results: 10 most significant negative everyday PD impacts.

**Figure 3:**
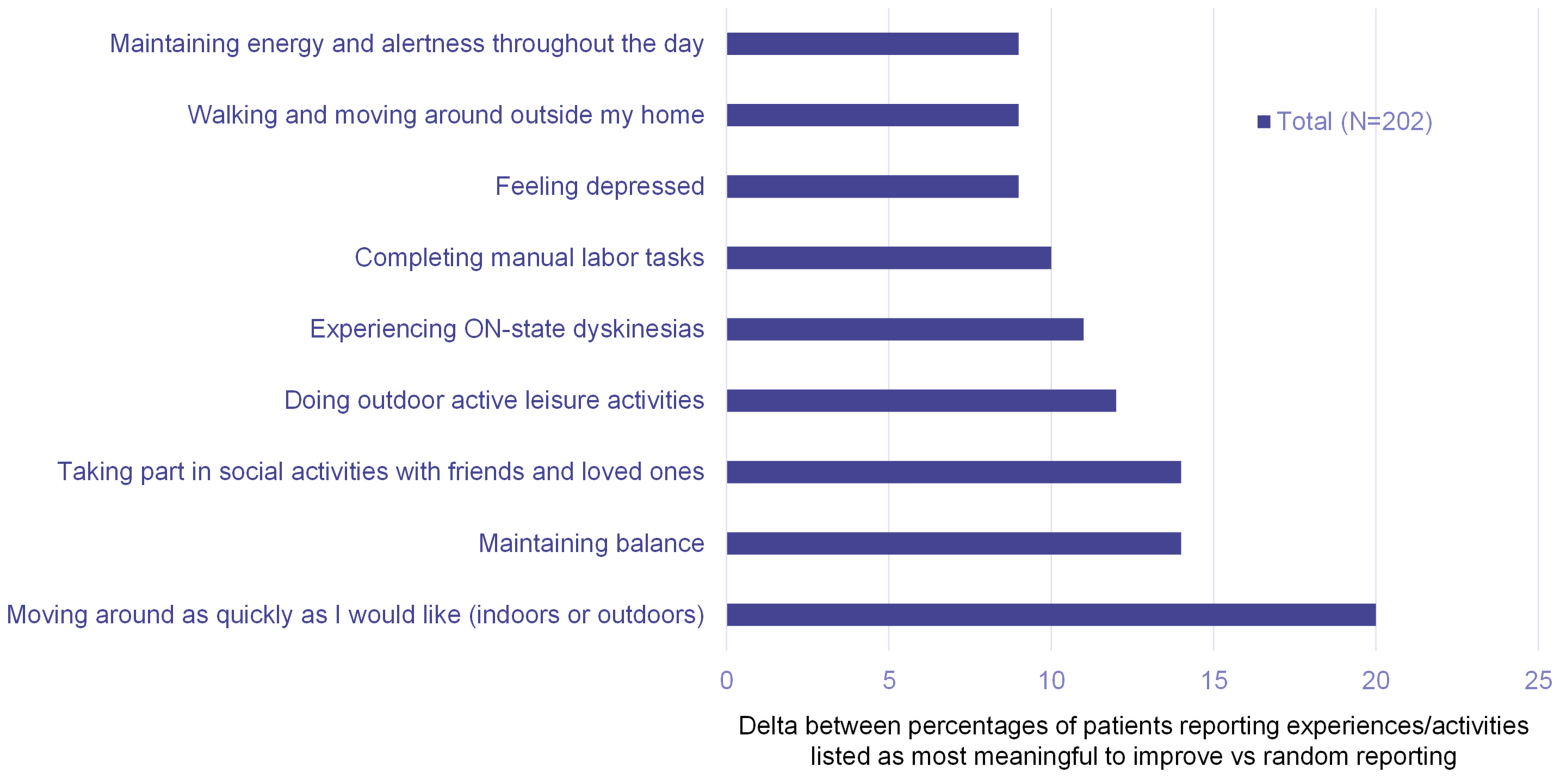
Patient online survey results: delta rankings of most meaningful experiences/activities to improve for people with iPD and *GBA1*-PD. Delta values represent the percentage of patients that reported the item minus the random reporting level (12.5% for eight choices).

In the survey, four of the top five most significant negative everyday PD impacts were related to motor symptoms (‘doing outdoor active leisure activities', ‘moving around as quickly as I like', ‘completing manual labor tasks' and ‘maintaining balance'); the other top five item was ‘feeling depressed' (Figure 2). Similarly, four of the top five most meaningful experiences/activities to improve for people with PD were related to motor symptoms (‘moving around as quickly as I like', ‘maintaining balance', ‘doing outdoor active leisure activities' and ‘experiencing ON-state dyskinesias); the other top five item was ‘taking part in social activities with friends and loved ones' (Figure 3).

### 3.4 Comparison of clinician- and patient-perspectives

According to the clinical advisory board meetings and patient surveys, highly meaningful symptoms and functional impacts were balance and gait difficulties, bradykinesia, global motor function difficulties, hands motor function difficulties, movement and mobility difficulties, rigidity/stiffness, and tremor (Table 2). Depression and fatigue were scored highly meaningful by patients but not clinicians.

**Table 2:** Meaningfulness classifications of PD symptoms and functional impacts rated by patients and clinical experts.

**PD functional aspect**	**Patient survey^a^**	**Clinical advisory board^b^**
**Non–motor**		
Anxiety	Medium	Medium
Bladder/bowel issues	Low	Low
Cognitive impairment: decision-making	Medium	Medium
Cognitive impairment: memory	Medium	Medium
Depression	High	Medium
Difficulties falling or staying asleep	Medium	Medium
Fatigue and energy issues	High	Medium
Hallucinations or delusions	Low	Medium
Overeating or undereating	Low	Low
Pain^c^	Medium	Medium
Sexual dysfunction difficulties	Medium	Low
**Motor**		
Balance and gait difficulties^d^	High	High
Bradykinesia^c^	High	High
Excessive drooling	Low	Low
Global motor function difficulties	High	High
Hands motor function difficulties	High	High
Movement and mobility difficulties	High	High
Rigidity/stiffness^c^	High	High
Speaking difficulties	Medium	Low
Standing or sitting difficulties	Low	Medium
Tremor^c^	High	High
**Dyskinesias and Dystonias**		
OFF–state dystonias	Medium	Medium
ON–state dyskinesias	Medium	High

^a^PD functional aspect was classified as low if it was consistently one of the least impactful and meaningful to improve across all relevant survey questions, medium if it was of average impact or meaningfulness to improve in all or some of the relevant survey questions, or high if it was consistently one of the most impactful and meaningful to improve across all relevant survey questions.

^b^PD functional aspect was classified as low if it was not mentioned, medium if it was mentioned but never explicitly discussed as highly face valid or important, or high if it was explicitly discussed as highly face valid and important.

^c^This aspect also prominently contributes to other PD aspects, especially hands and global motor function, movement/mobility, and gait/balance.

^d^This includes difficulties with diminished or varying stride length.

## 4 Discussion

Current scales that are designed to measure PD symptoms and severity are limited in that they require patients to visit clinics, regardless of their current condition and despite the fluctuating nature of PD symptoms (5). As part of our comprehensive approach to develop an at-home PD measurement instrument (PD-FIDI), we evaluated clinician- and patient-perspectives to determine which aspects of PD are most relevant and meaningful to patients' everyday lives in our target population. We found that the cardinal motor symptoms of PD (bradykinesia, rigidity, and tremor) and related mobility and hand-movement impacts were consistently cited as highly meaningful by clinicians and patients. Patients also considered select non-motor symptoms (pain, depression, and fatigue) to be highly meaningful.

In accordance with previous studies using different methodologies, the present results confirm that motor symptoms have significant negative impacts on the everyday lives of people with mild to moderate PD. Several studies have modeled the relationship between specific aspects of PD and quality of life (31–34), with a large analysis (*n* = 3,206) showing that MDS-UPDRS Part II (motor experiences of daily living) is the best predictor of quality of life of all the MDS-UPDRS parts (31). Our survey of people with mostly mild to moderate stages of PD supports and extends this work by identifying the specific negative everyday impacts of PD and those which people with PD view as most meaningful to improve. We found that motor symptoms and impacts directly underscore seven of the top 10 ‘significant negative everyday PD impacts' and six of the top 10 ‘most meaningful experiences/activities to improve'. The importance of bradykinesia (e.g, ‘moving around as quickly as I like') and tremor (e.g., ‘writing with a pen or pencil') is particularly evident. Of the qualitative research articles reviewed herein, the patient interviews performed by Politis et al. and Bonner et al. likewise identified bradykinesia and tremor as highly meaningful, and the research priorities determined by Deane et al. include tremor management (26–28). Additionally, patient interviews conducted as part of a study evaluating smartwatch and smartphone digital measures (WATCH-PD study), published during the preparation of this article, reported bradykinesia, tremor, and fine motor difficulties as the most bothersome and important symptoms for people with early PD (35). Therefore, when considered with previous findings, our results highlight that accurate and objective measurement of bradykinesia and tremor is essential for reliable monitoring of PD functional impact and treatment responses.

Our qualitative analyses of clinician and patient perspectives also considered the non-motor symptoms of PD which occur at all stages (36) and adversely impact quality of life (31–33). Accordingly, the research priorities of Deane et al. feature several non-motor impacts, including mental health, sleep, cognitive impairment, and urinary issues (27). The study by Politis et al. reported that non-motor symptoms generally have a greater impact at advanced versus early PD (26), which might be related to the increased prevalence and severity of non-motor symptoms at later disease stages (36, 37). In our survey of people with mild to moderate PD, depression (‘feeling depressed') and fatigue (‘maintaining energy and alertness throughout the day') were the only non-motor symptoms that ranked in the top 10 ‘most meaningful experiences/activities to improve' and ‘significant negative everyday PD impacts'. In addition to depression and fatigue, cognitive impacts were identified as highly meaningful by Bonner et al. (28), likely representing the higher proportion of participants in their study with *GBA1*-PD (75% versus 6% in our survey), which carries an increased risk of cognitive decline (38–40).

Depression and fatigue were considered significantly more meaningful by patients than clinicians in our study. In line with this observation, previous studies have reported that clinicians underdiagnose non-motor symptoms, including depression and fatigue (41), and view emotional wellbeing as less detrimental to quality of life than people with the condition (34). The discordant recognition of the impact of depression and fatigue in PD by patients and clinicians underscores the importance of patient-reported outcome measures in clinical practice and research.

Insights from the present study have informed the development of the PD-FIDI. We sought to incorporate aspects of PD into the PD-FIDI that were considered most meaningful by both patients and clinicians in our analysis. The device's ePRO component has been adapted to assess daily motor impacts and dyskinesia. Hand-based and global motor functional impacts, gait and balance, physical activity and general mobility are measured with app-based assessments, a wrist-worn wearable accelerometer device, or both (Figure 1). For some aspects of PD cited as meaningful by patients or clinicians in our study, the decision of how or whether to incorporate them into the PD-FIDI followed careful consideration of whether their assessment method is compatible with at-home digital sensor measurement; for example, app-based assessment of balance in the PD-FIDI excluded a sit-stand task due to the risk of falls, while depression was not incorporated because of concerns regarding the validity of longitudinal measurements using self-report scales (42, 43). It is acknowledged that the focus of our research on motor symptoms means that the omission of non-motor symptoms and impacts from the digital toolkit may require alternative methods of capturing fatigue and depression. It may also be the case that digital measures may help researchers and clinicians better monitor non-motor aspects and impacts, particularly sleep, fatigue, and depression, when used in conjunction with vital sign measures [e.g., cardiorespiratory signals for sleep (44)] and other assessments such as questionnaires.

Digital measurement tools involving smartphones and wearable devices are expected to facilitate clinical trials of potential PD therapies by increasing the objectivity, repeatability, accessibility, and convenience of rating, while substantially lowering the cost of motor symptom assessment (5). Moreover, since these tools can be administered more often than in-clinic PD assessments, their use in future PD trials may enable high levels of statistical power with relatively few participants (45). A smartphone and wrist-worn wearable was used to assess several exploratory endpoints in a Phase 2 trial of prasinezumab for early PD (46); initial results showed that adherence to the digital instrument was generally high over the 2-week baseline period, supporting the feasibility of in-home digital measurement in future PD clinical trials (47).

A limitation of our research is that primary-source patient perspectives were acquired through a voluntary online patient survey, which carries the risk of sampling bias. Additionally, most participants had mild to moderate PD, and our results may not be generalizable for people with advanced PD. However, people with less advanced PD are most likely to encounter digital endpoint instruments in future clinical trials, considering that early disease modification is widely viewed as having the best chance of success (48, 49). The survey sample was not perfectly representative, however, this work expanded upon earlier patient-perspective research conducted by Sanofi, by capturing patient input from a broader, more diverse, and closer to representative US patient population.

Finally, a limitation of the PD-FIDI is that it does not assess all highly meaningful aspects of PD that were identified in our qualitive research, with memory and decision-making not incorporated due to various technical reasons that rendered their assessment with PD-FIDI incompatible or impractical.

In conclusion, we evaluated patient and clinician perspectives to identify the most meaningful aspects of PD to be assessed with a digital endpoint instrument (the PD-FIDI). We are currently performing a clinical validation study to confirm the suitability of the PD-FIDI as digital measurement tool for PD symptoms and disease progression. Once validated, the PD-FIDI could be used to track PD symptoms more objectively in a less burdensome and obtrusive manner from the person's home environment, enabling physicians and sponsors of clinical trials to assess treatment responses with greater accuracy and ecological validity.

## Data Availability

The original contributions presented in the study are included in the article/Supplementary material, further inquiries can be directed to the corresponding author. Qualified researchers may request access to study data and related documents. Any patient level data will be anonymized, and study documents will be redacted to protect the privacy of the participants. Further details on Sanofi's data sharing criteria and process for requesting access can be found at: https://www.vivli.org.

## Author's note

Some of the data included in this manuscript were presented previously at the 7^th^ congress/2^nd^ virtual congress of the European Academy of Neurology (EAN), 19–22 June 2021 (Pearlmutter et al. EPO-637).
