# Supplementary material for: Clinician and patient perspectives on meaningful Parkinson's disease impacts for digital assessment
Source: Front Neurol. 2026 Mar 20;17:1677680. doi: 10.3389/fneur.2026.1677680 (PMC13048067; doi:10.3389/fneur.2026.1677680)
Supplement: Supplementary file 1 [file Table_1.docx]

**Supplementary Table 1.** Common everyday experiences and activities impacted by PD and included in the online survey.

| Feeling depressed | Holding utensils/glassware |
| --- | --- |
| Doing outdoor active leisure activities | Texting or using apps on a smartphone |
| Moving around as quickly as I would like (indoors and outdoors) | Experiencing OFF-state dyskinesias |
| Completing manual labor tasks | Completing personal hygiene tasks |
| Maintaining energy and alertness throughout the day | Carrying objects |
| Maintaining balance | Tying shoelaces |
| Taking part in social activities with friends and loved ones | Doing indoor leisure activities |
| Writing with a pen or pencil | Washing, drying & dressing yourself |
| Engaging in sexual activities | Staying focused & paying attention to details with decisions |
| Walking and moving around outside my home | Taking photographs |
| Falling or staying asleep | Forgetting names, places, or key items |
| Climbing upstairs | Maintaining bladder control |
| Driving a car | Standing up |
| Completing household chores | Maintaining regular bowel movements |
| Feeling anxious | Involuntary drooling during the day or while sleeping |
| Typing on a laptop or desktop | Experiencing hallucinations |
| Experiencing ON-state dyskinesias | Sitting down |
| Feeling dizziness when standing | Talking |
| Going downstairs | Not eating too much or too little |
| Walking and moving around inside my home | Chewing/swallowing food or drinks |

**Supplementary Table 2.** Final prioritized and ranked uncertainties for the management of PD. Table from “Priority setting partnership to identify the top 10 research priorities for the management of Parkinson's disease” by Deane, Flaherty (27), et al. Available at https://doi.org/10.1136/bmjopen-2014-006434, licensed under CC BY-NC 4.0.

| **Overarching research aspiration: an effective cure for Parkinson's disease** | |
| --- | --- |
| 1 | What treatments are helpful for reducing balance problems and falls in people with Parkinson's? |
| 2 | What approaches are helpful for reducing stress and anxiety in people with Parkinson's? |
| 3 | What treatments are helpful for reducing dyskinesias (involuntary movements, which are a side effect of some medications) in people with Parkinson's? |
| 4 | Is it possible to identify different types of Parkinson's, e.g., tremor dominant? And can we develop treatments to address these different types? |
| 5 | What best treats dementia in people with Parkinson's? |
| 6 | What best treats mild cognitive problems such as memory loss, lack of concentration, indecision and slowed thinking in people with Parkinson's? |
| 7 | What is the best method of monitoring a person with Parkinson's response to treatments? |
| 8 | What is helpful for improving the quality of sleep in people with Parkinson's? |
| 9 | What helps improve the dexterity (fine motor skills or coordination of small muscle movements) of people with Parkinson's so they can do up buttons, use computers, phones, remote controls etc.? |
| 10 | What treatments are helpful in reducing urinary problems (urgency, irritable bladder, incontinence) in people with Parkinson's? |

**Supplementary Table 3**. In depth concept elicitation interviews: patient-reported ‘most bothersome symptoms’ and ‘symptoms most important to treat. Table from “Patients' experiences of Parkinson's disease: a qualitative study in glucocerebrosidase and idiopathic Parkinson's disease” by Bonner, Bozzi (28), et al. Available at https://doi.org/10.1186/s41687-020-00230-9, licensed under CC BY 4.0.

| **Symptom** | **Number of patients who considered the symptom among the most bothersome or important to treat (≥5 *GBA1*-PD patients)^a^** | | |
| --- | --- | --- | --- |
|  | ***GBA1*-PD,  n/N (%)** | **iPD,  n/N (%)** | **Total,  n/N (%)** |
| Fatigue/tiredness | 14/15 (93%) | 5/5 (100%) | 19/20 (95%) |
| Tremor | 13/15 (87%) | 5/5 (100%) | 18/20 (90%) |
| Memory loss | 13/15 (87%) | 5/5 (100%) | 18/20 (90%) |
| Walking limitations | 12/15 (80%) | 5/5 (100%) | 17/20 (85%) |
| Bradykinesia/slowness | 12/15 (80%) | 5/5 (100%) | 17/20 (85%) |
| Pain | 12/15 (80%) | 5/5 (100%) | 17/20 (85%) |
| Urinary problems | 12/15 (80%) | 5/5 (100%) | 17/20 (85%) |
| Balance/postural instability | 12/15 (80%) | 4/5 (80%) | 16/20 (80%) |
| Rigidity/stiffness | 11/15 (73%) | 5/5 (100%) | 16/20 (80%) |
| Limb weakness | 11/15 (73%) | 5/5 (100%) | 16/20 (80%) |
| Orthostatic hypotension | 11/15 (73%) | 5/5 (100%) | 16/20 (80%) |
| Attentional impairments | 10/15 (67%) | 4/5 (80%) | 14/20 (70%) |
| Cramps | 10/15 (67%) | 4/5 (80%) | 14/20 (70%) |
| Constipation | 9/15 (60%) | 4/5 (80%) | 13/20 (65%) |
| Speech problems | 11/15 (73%) | 1/5 (20%) | 12/20 (60%) |
| Tingling numbness | 9/15 (60%) | 1/5 (20%) | 10/20 (50%) |

*GBA1*-PD, people with Parkinson’s disease and glucocerebrosidase variant; iPD, idiopathic Parkinson’s disease. ^a^Determined by asking the patients to identify the three symptoms they felt were most bothersome and most important to be treated.

**Supplementary Table 4**. Demographics and disease characteristics of the population for the PD patient online survey.

| **Characteristic** | **All Participants (N=202)** |
| --- | --- |
| Age (years), mean | 49 |
| Male, n (%) | 119 (58.9) |
| iPD, n (%) | 189 (93.6) |
| GBA-PD, n (%) | 13 (6.4) |
| Ashkenazi Jewish ancestry, n (%) |  |
| None | 138 (68.3) |
| Partial | 14 (6.9) |
| Full | 33 (16.3) |
| Unknown | 17 (8.4) |
| H&Y Scale stage, n (%) |  |
| 1 or 1.5 | 13 (6.4) |
| 2 or 2.5 | 80 (39.6) |
| 3 | 76 (37.6) |
| 4 | 29 (14.4) |
| 5 | 4 (2.0) |

*GBA1*-PD, people with Parkinson’s disease and glucocerebrosidase variant; H&Y, Hoehn and Yahr; iPD, idiopathic Parkinson’s disease.
